# 

**Published:** 1885-04

**Authors:** 


| | PAGE |
|---|---|
| ORIGINAL COMMUNICATIONS.—Robert Koch, the Conductor of the German Cholera Commission, and his Method of Bacteria Cultivation. Prof. Dr. Albert Johne | 113 |
| The Physiological Action of Cocaine on the Frog. H W. Biggs, M.D | 128 |
| Tuberculosis in the Udder of the Milch Cow, and its Effects upon the Milk. Dr B. Bang | 143 |
| Azoturia. Thomas W. Rogers, V.S | 156 |
| Sulphuretted Hydrogen as a Product of Indigestion, and its Action upon the System. John A. McLaughlin, D.V.S | 162 |
| Pulmonary Emphysema and its Relation to Heaves in the Horse. Prof. Wilhelm Dieckerhoff | 68 |
| The History of Tuberculosis. Dr. Albert Johne | 180 |
| EDITORIAL.—The Relation of General Pathology to Medical Science.—The Bureau of Animal Industry.—Appointment of Dr. Billings.—Toronto Veterinary College.—Notes | 189 |
| OBITUARY | 201 |
| CASE DEPARTMENT.—Autopsy at Central Park Menagerie.—Two Cases of Azoturia.—Radical Cure of External Pulmonary Hernia | 201 |
| PROCEEDINGS OF VETERINARY MEDICAL SOCIETIES.—American Veterinary College.—Ontario Veterinary College.—Act to Incorporate Veterinary Society | 204 |
| CORRESPONDENCE | 205 |
| REVIEWS.—The Anatomy of the Horse.—Manual of the Theory and Practice of Equine Medicine.—Guernsey Breeders Journal.—Therapeutic Gazette.—Books and Pamphlets received | 206 |
| NEWS AND MISCELLANY | 209 |
| PROGRESS OF VETERINARY SCIENCE | 214 |

